# How the Green Architecture of the 2023–2027 Common Agricultural Policy could have been greener

**DOI:** 10.1007/s13280-023-01861-0

**Published:** 2023-05-06

**Authors:** Herve Guyomard, Cécile Détang-Dessendre, Pierre Dupraz, Luc Delaby, Christian Huyghe, Jean-Louis Peyraud, Xavier Reboud, Clélia Sirami

**Affiliations:** 1 INRAE, SDAR Centre Bretagne-Normandie, Domaine de la Motte, BP 35327, 35653 Le Rheu Cedex, France; 2 INRAE, UMR CESAER, 26 Boulevard Docteur Petitjean, BP 87999, 21079 Dijon Cedex, France; 3 INRAE, UMR SMART, 4 allée Adolphe Bobierre, CS 61103, 35011 Rennes Cedex, France; 4 INRAE, UMR PEGASE, 16 le clos (Ex-Domaine de la Prise), 35590 Saint-Gilles, France; 5 INRAE, CODIR, 147 rue de l’université, 75338 Paris Cedex 07, France; 6 INRAE, UMR Agroecology, 17 rue Sully, BP 86510, 21065 Dijon Cedex, France; 7 INRAE, UMR DYNAFOR, 24 Chemin de Borde Rouge, CS 52627, 31320 Auzeville-Tolosane, France

**Keywords:** Agri-environment and climate measures (AECM), Common Agricultural Policy (CAP), Conditionality, Eco-schemes, Environment, European agriculture

## Abstract

A new 5-year Common Agricultural Policy has been in place since January 2023. Like its predecessors, this new policy will fail to deliver significant climatic and environmental benefits. We show how the Green Architecture of the policy relying on the three instruments of conditionality, eco-schemes, and agri-environment and climate measures could have been used more consistently and effectively. Our proposals are based on core principles of public economics and fiscal federalism as well as on research results in agronomy and ecology. Conditionality criteria are the minimal requirements that every agricultural producer must meet. Farmers should be rewarded for efforts that go beyond these basic requirements through eco-schemes for global public goods complemented by agri-environment and climate measures centred on local public goods. Eco-schemes should cover the whole agricultural area by targeting permanent grasslands, crop diversification, and green cover and non-productive agro-ecological infrastructures. We discuss trade-offs that our proposals could generate.

## Introduction

The climate and environmental emergencies have been documented (Pörtner et al. 2021). In the European Union (EU), it has led the European Commission (EC) to launch the European Green Deal (EGD) in December 2019 (EC 2019). The EGD encompasses all sectors, including agriculture and food through the Farm to Fork Strategy (EC 2020a) and the EU Biodiversity Strategy for 2030 (EC 2020b).

Negotiations on the future Common Agricultural Policy (CAP) were initiated before the launching of the EGD. After more than 3 years of conflictual discussions, the European Council, Parliament, and Commission reached a political agreement in June 2021 for the next CAP, which will be applied for 5 years from 1 January 2023. According to the EC, this new CAP will be “greener” (EC 2021a). The new policy includes provisions that support this assertion. However, these provisions are likely not up to the challenge and the EGD ambition. In that context, this paper proposes more ambitiously greening the CAP, building on the climatic and environmental instruments of the Green Architecture (GA) of the June 2021 agreement, i.e. conditionality requirements, eco-schemes, and agri-environment and climate measures (AECM).

The paper is organized as follows. "Climate and environment in the 2023–2027 CAP" section summarizes the June 2021 CAP agreement and underlines its insufficient climatic and environmental ambition. "Defining Pillar 1 eco-schemes in articulation with Pillar 2 climate and environment measures" section shows how it would be possible to use this agreement's instrumental framework to increase the CAP's climatic and environmental ambition. The proposal is based on simple principles of public economics and fiscal federalism as well as on research results in agronomy and ecology. "Three issues to be addressed" section discusses three key issues that can undermine the climatic and environmental ambition of the CAP, i.e. (i) the difficulty in quantifying climatic and environmental impacts of policy measures, (ii) the existence of possible trade-offs between competing objectives, and (iii) the trade dimension discussed here in the light of both the compliance with World Trade Organization (WTO) rules and pollution leakages. We conclude by placing the CAP analysis in the context of the COVID crisis and the war in Ukraine, positioned in the broader perspective of food systems central to the EGD.

## Climate and environment in the 2023–2027 CAP

### The Green Architecture of the 2023–2027 CAP

**Fig. 1:**
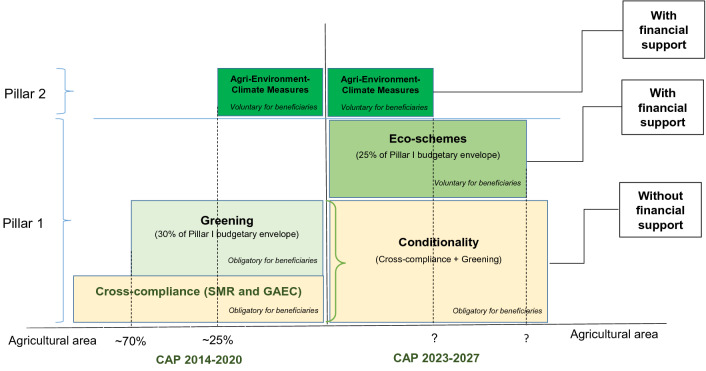
The Green Architecture of the 2014–2020 and 2023–2027 CAP. *Source* Own elaboration from EC (2017), Lotz et al. (2019), EC (2021a, 2021b), Official Journal of the EU (2021), and Pe’er et al. (2022)

The 2023–2027 CAP includes three instruments devoted to climate mitigation and environmental protection (Fig. 1). Two instruments were already in place in the previous CAP, i.e. conditionality and AECM. New conditionality requirements encompass cross-compliance criteria and the three greening measures of the 2014–2020 CAP. Cross-compliance sets basic rules farmers must comply with to receive income support direct aids of Pillar 1, with penalties in case of non-compliance. These rules include statutory management requirements (SMR) on the environment, health, and animal welfare and norms aimed at keeping land in good agricultural and environmental conditions (GAEC). Cross-compliance is extended by including the three measures of the green payment of the 2014–2020 CAP related to the preservation of permanent grasslands, a minimal diversification of annual crops, and the maintenance of Ecological Focus Areas (EFA). Eco-schemes are a novel instrument that shares several features with AECM (Table 1). Like the latter, eco-schemes must address climate and environment objectives that go beyond conditionality requirements and are compulsory for member states (MS) but optional for farmers (both instruments can also address animal welfare issues). Unlike AECM, which are a Pillar 2 instrument and are thus co-funded by national and regional authorities, the European budget fully funds eco-scheme payments of Pillar 1. These payments will be granted per hectare in two forms, i.e. i) like AECM in compensation for additional costs incurred or income foregone induced by more environmentally friendly agricultural practices or ii) unlike AECM, as fixed top-up payments in addition to decoupled direct payments referred to as basic income support for sustainability (BISS) in the 2023–2027 CAP. However, if an eco-scheme targets production of a specific crop or prescribes a particular agricultural land use, only the cost incurred/income foregone option i) can be applied to ensure the classification in the green box of the WTO (EC 2021b). Eco-scheme payments will be granted annually, although the June 2021 agreement allows MS to offer multiannual payments. Two climatic and environmental budgets are ring-fenced, with 25% of Pillar 1 direct payments allocated to eco-schemes and 35% of Pillar 2 budgetary envelope devoted to AECM and payments for areas with natural constraints.

**Table 1:** Comparison of CAP climatic and environmental instruments: eco-schemes *versus* AECM

	Eco-schemes	AECM
Focus	Climate and environment	Climate and environment
Mandatory/voluntary	Mandatory for MS, optional for farmers and land managers	Mandatory for MS, optional for farmers
Funding	Pillar 1 (EU budget)	Pillar 2 (EU and MS budgets)
Payment basis	Per hectare	Per hectare
Support calculation	Compensation for costs induced or income loss or Fixed top-up payment to decoupled payments (BISS)	Compensation for costs induced or income loss
Commitment period	Annual or multiannual	Multiannual (usually around 5 years)
Minimum spending requirement	At least 25% of the pillar 1 budget	At least 35% of the pillar 2 budget devoted to climatic and environmental measures of this pillar, including AECM

*Source* Own elaboration from EC (2017), Lampkin et al. (2020), Guyomard et al. (2020), EC (2021a, 2021b), Matthews (2021), Official Journal of the EU (2021)

### A climatic and environmental ambition for a large part in the hands of member states

The initial CAP reform proposals presented by the EC in June 2018 claimed that a higher level of climatic and environmental ambition was imperative. The climatic and environmental challenges of EU agriculture are well documented, as well as the failure of the CAP to address them so far (Pe’er et al. 2019; Guyomard et al. 2020).

The green payment of the 2014–2020 CAP has notably been heavily criticized for its very low environmental effectiveness (European Court of Auditors—ECA 2017). Including greening measures into conditionality requirements of the 2023–2027 CAP obeys the no-backsliding principle (Table 2). Furthermore, new requirements have been added as part of either SMR (for example, by including some elements of the Water Framework Directive and the Directive on the Sustainable Use of Pesticides) or GAEC (through new GAEC 2 requiring MS to protect wetlands and peatlands, revised GAEC 4 imposing buffer strips of at least 3 m, the emphasis of GAEC 7 on crop rotation with greater environmental benefits than crop diversification, and an increased focus of GAEC 8 on non-productive areas).

**Table 2:** Comparison of cross-compliance and greening obligations in the 2014–2020 CAP and conditionality requirements in the 2023–2027 CAP

2014–2020 CAP	2023–2027 CAP
*SMR*	
SMR	Same SMR + New SMR related to the Water Directive, the Drinking Water Directive, the Framework for the sustainable use of pesticides, the Plant Protection Products Regulation, the Directive and Regulation on veterinary medicinal products
*GAEC and greening measures (GM)*	
Climate change	
GM 2: Maintenance of permanent grassland according to the ratio of permanent grassland to agricultural area (decline limited to 5% max.)	GAEC 1: Same requirements except i) new reference year (2018 instead of 2012) and ii) inclusion of organic farmers who were previously exempted
	GAEC 2: New; appropriate protection of wetland and peatland
GAEC 6: Interdiction of burning arable stubble except for plant health reasons	GAEC 3: Same requirements
*Water*	
GAEC 1: Establishment of buffer strips along water courses	GAEC 4: Same requirements (ban on nutrients’ and pesticides’ uses integrated in the GAEC text)
GAEC 2: Compliance with authorisation procedures where irrigation is subject to authorisation	
GAEC 3: Protection of groundwater against pollution	
*Soil protection and quality*	
GAEC 5: Tillage management in order to reduce the soil degradation	GAEC 5: Same requirements
GAEC 4: Minimum soil cover	GAEC 6: No bare soil in most sensitive periods
GM 1: Minimal crop diversity on arable areas with three exemptions (small farms of less than 10 ha, organic farms, farms essentially in grass or rice); notions defined in EU Regulation 1307/2013	GAEC 7: Crop rotation or equivalent practices, including through crop diversity; possibility to define the rotation at the scale of the crop year (main crop/intermediate crop); same exemptions; notions to be defined in national strategic plans (NSP)
*Biodiversity and landscapes*	
GAEC 7: Maintenance of landscape figures; interdiction of cutting trees and hedges during the breeding and nesting season of birds GM 3: Implantation of EFA on at least 5% of the arable land area	GAEC 8: Establishment of non-productive EFA on at least 4% of the arable land area (option 1) or on at least 7% if the MS decides to include productive elements (catch crops, nitrogen-fixing crops) with then at least 3% on non-productive elements (option 2); reduction of the requirement of the first option to 3% of arable land if the farmer enrolled in an eco-scheme agrees to increase the area allocated to non-productive areas to 7% or more
GM 2: In Natura 2000 zones, ban on ploughing or converting permanent grassland	GAEC 9: Same requirements

*Source* Adapted from EC (2017), Matthews (2018), Dupraz and Guyomard (2019), EC (2021a), Official Journal of the EU (2021). The table defines the correspondences between GAEC of the 2023–2027 CAP (right column) and GAEC and GM of the 2014–2020 CAP (left column)

Other provisions contribute to greening the new policy. The 25% ring-fencing in Pillar 1 is a step towards repurposing income support direct payments. The 35% ring-fencing in Pillar 2 is a significant increase over the 30% in the previous CAP given that payments for areas with natural constraints now only contribute 50% to this total. A change in the definition of ‘eligible hectares’ allows CAP payments for scrubs and non-productive features without necessarily clearing them as in the previous CAP. The policy provides greater flexibility to ensure that climatic and environmental targets of both eco-schemes and AECM are met.

However, the climate and environmental ambition of the new CAP cannot be truly appreciated as long conditionality implementation choices by the different MS are not known in detail. For example, the previous greening criterion on crop diversification is now replaced by GAEC 7 on crop rotation, which has greater benefits for soil health. However, MS can still opt for the crop diversification criterion provided they can demonstrate that this alternative “clearly helps to preserve soil potential” (EC 2021a). In addition, as in the previous CAP, farms with less than 10 hectares (ha) of arable crops or with a large proportion of permanent grassland are exempted from GAEC 7 requirements, and organic holdings are automatically considered as meeting them.

What is true for conditionality is even truer for eco-schemes and AECM since the June 2021 CAP agreement only provides general funding rules (two ring-fenced budgets) and principles (supporting voluntary actions that go beyond minimal conditionality requirements). The fact that eco-scheme payments will not necessarily be linked to costs incurred and/or income foregone when MS grant them as top-up payments to BISS is a first step towards payments for climate and environment services (Guyomard et al. 2020). However, nothing constrains a MS from explicitly linking eco-scheme payments to ecological services delivered. Indeed, the June 2021 decisions only state that eco-schemes must reward farmers for implementing climate and environmentally friendly agricultural practices. Like most AECM, eco-schemes will mainly rely on prohibited and/or required farming practices rather than on results and a fortiori impacts. The June 2021 decisions are silent on measures within Pillar 2. They only comment on their simplification by consolidating the 20 measures and 64 sub-measures previously used into eight broad types and interventions (EC 2021a). In particular, they do not provide guidelines to increase the ecological efficiency of AECM by correcting their main drawbacks linked to public and private transaction costs, windfall effects, and temporal and spatial discontinuity of ecological targets (Cullen et al. 2018; Dupraz and Guyomard 2019).

This means that the climatic and environmental ambition and efficiency of the 2023–2027 CAP depend on how MS will implement the three green architecture instruments in their respective national strategic plan (NSP). The latter describes the intervention logic each MS uses to address its priority needs and contribute to the nine objectives of the CAP, including the three objectives related to climate change mitigation and adaptation, the sustainable use of natural resources (water, soil, and air), and the protection of biodiversity. This raises questions about the administrative and technical capacity of MS to prepare high-quality NSP (Ervajec 2020). This also raises questions about their political will to propose NSP that will be sufficiently ambitious from a climatic and environmental point of view and to resist farm and food lobby groups that are favourable to the status quo on the grounds of competitiveness and income arguments.

Extending the approach already used for Pillar 2, the EC must approve NSP and control their implementation and outcomes in the CAP new delivery model (NDM) framework based on context, output, result, and impact indicators. This performance-focussed NDM can be viewed as a step in the right direction. However, its ability to achieve a high level of climatic and environmental ambition can be questioned for several reasons. First, because the reasons explained above might lead to a race to the bottom under the pretext of avoiding competitiveness distortions among MS. Second, because of poor accountability mechanisms between measures and objectives, the incompleteness of performance indicators that will be used to measure progress, as well as too vague monitoring, reporting, and evaluation procedures at both the MS and EU levels (ECA 2018; Heyl et al. 2020; Pe’er et al. 2022). Furthermore, there are doubts about the political ability of the EC to request changes in a given NSP if it judges the latter as insufficiently ambitious and if results are not achieved over the 2023–2027 period. Preliminary analyses of first drafts of NSP and eco-schemes suggest that these are not ambitious from a climatic and environmental point of view (EEB-BirdLife International, 2022; Runge et al*.*, 2022).

## Defining Pillar 1 eco-schemes in articulation with Pillar 2 agri-climate and environment measures

### Agricultural practices that should be supported through eco-scheme measures

The EC states that “eco-schemes should cover activities related to climate, environment, animal welfare and antimicrobial resistance” for efforts going beyond minimal conditionality requirements and prioritized based on an assessment of national/regional needs” (EC, 2021c). These criteria also apply to AECM in Pillar 2. It is, therefore, critical to clarify which measures should be included in the Pillar 1 eco-schemes *versus* Pillar 2 AECM. Following lessons of the theory of fiscal federalism (Oates 1972), eco-schemes that are totally funded by the EU budget should target the provision of global public goods, while AECM measures that are co-financed by national/regional authorities should be devoted to local environmental issues. Eco-schemes must thus primarily focus on reducing GHG agricultural net emissions and the preservation/restoration of biodiversity in agro-ecosystems.

The greening criteria of the 2014–2020 CAP aimed essentially at improving soil quality (greening measure on crop diversification), increasing carbon sequestration (permanent grasslands), and preserving and improving farmland biodiversity through the measure devoted to EFA. These criteria have been heavily criticized for their very low environmental effectiveness (ECA 2017), notably because of the large flexibility left to MS in implementing greening requirements that has significantly weakened the initial ambition of the Commission. Based on this experience and the content of the June 2021 CAP agreement, one cannot anticipate significant climate and biodiversity benefits from including greening criteria in the 2023–2027 CAP conditionality requirements. Greening requirements can, however, be used as reference requirements, on which it is possible to define more ambitious targets for practices that could be supported through eco-scheme payments. More specifically, requirements of the three greening measures included in the conditionality of the 2023–2027 CAP are here used as ‘starting levels’ to develop three eco-scheme measures covering the whole agricultural area thanks to practices related to i) permanent grasslands, ii) crop diversification and green cover, and iii) EFA. For each of these three measures, we propose a detailed description of practices that would entitle farmers to eco-scheme payments.

#### (a) Maintaining permanent grasslands over the long run

Permanent grassland in defined in Article 4 of the Regulation (EU) 2021/2115 on NSP (Official Journal of the EU, 2021). Except in Natura 2000 areas where GAEC 9 will continue to prevent the ploughing of permanent grassland, conditionality requirements allow this ploughing provided that an equivalent area is converted into permanent grassland with negative impacts on many ecological services.

The organic carbon storage service of non-ploughed permanent grassland increases with the age of the latter, over around 150 years (Smith 2014). Low-intensity long-term permanent grasslands are a hot spot of specific biodiversity and allow the intensive biological activity of soil microorganisms (Marriott et al. 2004). Permanent grasslands improve water resource quality by acting as biological filters that limit the migration of chemical molecules towards surface and groundwater systems (Jankowska-Huflejt 2006). They also reduce soil erosion and runoff thanks to a permanent soil cover and a dense rooting system (Cerdan et al. 2010).

Permanent grasslands with legumes provide additional ecological benefits since they enable symbiotic fixation of atmospheric nitrogen, reducing the need for nitrogen fertilization and associated nitrous oxide emissions (Klumpp et al. 2011). They are more resilient and productive (Barneze et al. 2020) and provide high-quality fodder that is well valued by livestock (Luscher et al. 2014).

For fixed climate and soil conditions, grassland management practices affect the ecological services they provide. Specific flora diversity and insect abundance decrease when livestock density increases (Wallis De Vries et al. 2007). The quality of water in sown grasslands, measured in terms of nitrogen and phosphorus flows or bacterial contamination, is affected by the grassland age and its management intensity (Vertès et al. 2010). The buffering role of grasslands established for only a few years is reduced.

Table 3 summarizes the previous analyses by defining a simplified qualitative assessment of grasslands' ecological benefits, which are differentiated according to age, composition (with *versus* without legumes), and management practices (animal density). This typology can be used to define a first eco-scheme measure/payment for permanent grasslands that would be granted only in case of no ploughing at the plot level. The payment level would be differentiated according to permanent grassland age (between 5 and 10 years, between 10 and 20 years, and older than 20 years). A first bonus would be granted for grasslands with legumes, and a second bonus when livestock density is lower than a threshold, say of 1.5 Gross Livestock Units (GLU) per hectare of grassland.

**Table 3:** Ecological benefits of grasslands in function of age, composition, and management practices

Grassland type	Carbon storage	Biodiversity	Soil (quality)	Water (quantity)	Water (quality)
Temporary grassland (TP) (< 5 years)					
TP without legumes	0	0	0	0	−/ +
TP with legumes	−/ +	+	+	0	+
Permanent grassland (PG) between 5 and 10 years (1)					
PG without legumes	+	0/ +	+	+	+
PG with legumes	+ / + +	+ / + +	+ / + +	+	+ +
Permanent grassland (PG) above 10 years					
Intensive PG (2)	+ + +	+ +	+ +	+ +	+ +
Extensive PG (3)	+ + +	+ + +	+ +	+ + +	+ + +

*Source* Own elaboration. (1) In a general way, ecological benefits of PG increases with the age of the latter; (2) Animal density > 1.5 GLU per ha of grassland; (3) Animal density < 1.5 GLU per ha of grassland

#### (b) Crop diversification and green cover

The second eco-scheme measure/payment aims at rewarding crop diversity beyond the minimal requirements of the crop diversification conditionality criterion of the 2023–2027 CAP. It is based on research results that have pointed out the negative ecological consequences of insufficient crop diversity (Kleijn and Sutherland 2003; Whittingham 2007). The payment would increase with the value of a crop diversity index based on a classification of crops gathering plant species that exhibit similar responses to biotic and abiotic pressures and have similar effects on the functioning of agro-ecosystems (Hector et al. 1999). As a result, such crop classification would be based on main botanical families (cereals, Brassicaceae, legumes), seeding periods (winter *versus* spring), duration of stay (annual *versus* perennial crops), and planting density (root crops *versus* other crops). Table 4 proposes such a classification for France. This classification must be adapted to each MS according to climatic conditions that largely determine crops that can be cultivated.

**Table 4:** Classification in eight functional groups of crops eligible to the crop diversification eco-scheme measure: illustration for France

Functional groups	Composition
1	Winter cereals
2	Spring cereals
3	Root plants (potato, beet, corn, sorghum, sunflower)
4	Winter oilseeds (winter rapeseed)
5	Spring oilseeds (spring rapeseed, mustard, flax)
6	Protein crops
7	Temporary grasslands, including alfalfa
8	Other crops: industrial perennial crops (miscanthus, silphie, etc.), aromatic and perfume plants (lavender, lavandin, clary sage, etc.), etc

*Source* Own elaboration. Intermediate crops implemented between main crops are not included for several reasons: first, for implementation simplicity of the measure; second, because they are covered through the bonus for green cover (see text); third, because implementing intermediate crops is closely linked to the presence of spring crops. In the same way, mixed crops such as wheat and pea are counted as one crop only

A first bonus would be granted to farms with an average plot size lower than 4 hectares because ecological benefits are greater on lower-size plots than on larger-size plots (Fahrig et al. 2015). A second bonus would be granted to farms maintaining permanent soil coverage because cover crops provide numerous ecological benefits, including increases in soil quality (organic matter, structure, and fertility), decreases in water and wind erosion, and increases in atmospheric nitrogen fixation when legumes are introduced (Wittwer et al. 2017). However, cover crops can present drawbacks when they limit water availability for the following crop or when they are destroyed using pesticides. As a result, NSP should carefully define the eligibility criteria for this second bonus, considering regional/local characteristics.

**Fig. 2:**
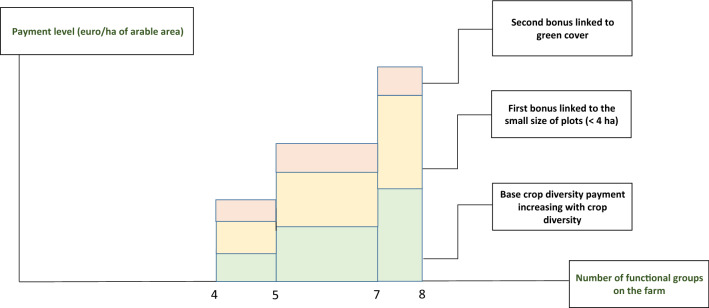
Operating principles of the crop diversification and green cover eco-scheme measure /payment. *Source* Own elaboration. For simplicity reasons, the x-axis of this figure represents the number of functional crop groups on the farm

Figure 2 summarizes the logic and the functioning of this crop diversification and green cover eco-scheme measure/payment.

#### (c) Non-productive areas

Among eligible EFA types within the 2014–2020 CAP, ecologists value field margins, buffer strips, fallow land, and landscape features as the most beneficial for biodiversity. This contrasts with farmers’ choices, mainly for catch crops, nitrogen-fixing crops, and fallow land. This largely explains the ecological failure of the EFA greening measure of the 2014–2020 CAP (Pe’er et al. 2016). Unlike the latter, GAEC 8 of the 2023–2027 CAP rightfully focuses on non-productive areas (Table 2).

The third eco-scheme measure/payment we propose is built on GAEC 8 by rewarding farmers for allocating more than 4% of their total agricultural area to non-productive uses. Three payment levels would be defined for non-productive areas covering between 4 and 7%, 7 and 10%, and above 10%. A first bonus/malus would encourage farmers to maintain and develop non-productive infrastructures that are relatively rare in the region where the farm is located for increased ecological benefits (Andersson et al. 2013). This could be achieved by differentiating weighting factors of non-productive areas at a regional scale, i.e. by increasing (respectively, decreasing) coefficients associated with relatively rare (abundant) infrastructures. A second bonus would aim at capturing the ecological benefits of non-productive areas increase with their spatial continuity requiring the joint and consistent involvement of a sufficient number of farms (Baudry et al. 2003; Krämer and Wätzold 2018). This agglomeration bonus would be designed at the same spatial scale used for the first bonus/malus for reducing implementation costs, simplicity, and consistency.

### Defining measures of the second pillar in a complementary and consistent way

The Pillar 2 AECM instrument is flexible. It is used to reach a great diversity of ecological targets. However, it is difficult to design, implement, administer, control, and assess (Cullen et al. 2018). Public and private administration costs are high. These costs could be reduced by increasing the contract duration over a period longer than the maximum of 7 years of the Multiannual Financial Framework. They could also be reduced by encouraging the spatial continuity of measures thanks to group subscriptions that could be rewarded through agglomeration bonuses. Both improvements would have the additional benefit of increasing ecological efficiency thanks to adopting measures at the most relevant ecological level, usually not the farm or the plot level. Westerlink et al*.* (2017) showed that AECM that constrain farmers to spatially coordinate their actions and/or pay farmers according to observable environmental impacts are ecologically and economically more efficient.

Unfortunately, the June 2021 CAP agreement does not introduce the possibility for AECM to remunerate ecological services beyond the compensation of costs induced or income foregone. Furthermore, AECM will likely continue to be mainly based on an obligation of practices. Links with positive ecological impacts will remain insufficiently quantified.

In line with fiscal federalism lessons, AECM should essentially sustain the provision of local public goods such as soil, water, air, and landscape quality. Frequently, they could be used to enhance eco-scheme measures in sensitive areas that face localized issues, for example, in water catchment areas where soil, air, and water quality are threatened by excessive use of pesticides and fertilizers. This means that a farmer should benefit from eco-scheme payments when she(he) respects criteria associated with eco-scheme requirements and be compensated through AECM for extra costs linked to additional efforts responding to local needs. The choice of farms and areas eligible for AECM should involve all relevant stakeholders (e.g. farmers, environmental associations, and regional councils). In addition, AECM should continue to support agricultural system changes, which currently suffer from low adoption rates. One can hope to increase the adoption rates of the system-AECM by rewarding some prohibited or mandatory practices with eco-scheme payments. This rule would notably apply to organic farmers who should benefit from the highest eco-scheme payments as defined above.

Moving away from a cost incurred/income foregone benchmark may, however, present drawbacks (Matthews 2021). The solution remains subject to political bargaining and rent seeking related to the calculation of ecological benefits and their monetary value. Furthermore, proportioning payments to ecological benefits could reduce their provision in a context of a limited ecological budget.

Criteria used for granting Pillar 2 investment aid for buildings and materials should be considerably strengthened by linking them to conditionality requirements, completed by additional criteria demonstrating that supported investment helps to reduce GHG emissions and/or the use of chemical inputs (pesticides, fertilizers, antibiotics). It would be more efficient to support precision farming in this way rather than through a specific eco-scheme flagship program (Pe’er et al. 2022). In the case of livestock buildings, cross-compliance criteria should encompass animal welfare. Finally, CAP payments for less favoured areas (LFA) introduced in the 1970s should continue to address an income compensation objective because farmers' incomes in disadvantaged areas are generally lower than those of their counterparts in plain areas. As noted by Dupraz and Guyomard (2019), “these payments are also justified on the ground that maintaining an agricultural activity [in disadvantaged areas] is beneficial for the environment because it limits farmland abandonment, maintains diversified landscapes, and preserves biodiversity”. On this point, the main issue is documenting the co-benefit for the environment.

## Three issues to be addressed

### Assessing the sustainability of CAP reform

Quantitatively assessing the climate and environmental benefits of any CAP reform remains a challenge, given the data, indicators, and integrated modelling frameworks that are currently available.

The successive CAP reforms implemented since 1992 have failed by being unable to reduce the ecological footprint of EU agriculture. This failure is well documented in numerous *ex post* analyses that pointed out the drawbacks of cross-compliance (ECA 2016), greening (ECA 2017), or AECM (Cullen et al. 2018). It is more than likely that this will also be the case with the June 2021 CAP agreement, even if this remains to be seen depending on national declinations of the latter in NSP. To conduct *in itinere* and *ex post* analyses of climate and environmental impacts of these national choices—it is unfortunately too late to develop *ex ante* assessments—each MS must implement a complete and robust quantitative assessment framework based on relevant and well-designed ecological impact indicators. Unfortunately, the set of indicators that will be used to implement, monitor, and control the NSP is unsatisfactory from this point of view (ECA 2018; Guyomard et al. 2020). Such an assessment framework should cover all sustainability dimensions to highlight trade-offs and establish corrective measures if needed. It should not be limited to the green architecture instruments but extended to relevant regulations and all CAP instruments with possible unintentional ecological impacts. Such a framework is lacking at both the MS and EU level. In the different NSP, the relationships between political measures and ecological impacts are often postulated (or hoped) rather than demonstrated. In that context, our proposals for eco-schemes and AECM presented in "Defining Pillar 1 eco-schemes in articulation with Pillar 2 climate and environment measures" section stand out because they are based on scientific evidence of practices with well-established positive ecological impacts. Moreover, they are consistent with lessons from public economics and fiscal federalism. This provides a solid theoretical framework to build policy measures whose efficiency can be tested through quantitative assessments.

### Explicitly addressing possible trade-offs between objectives

The 2023–2027 CAP includes nine specific objectives with three objectives on each sustainability dimension (EC 2017). No one will dispute their relevance, but this does not mean they are automatically compatible (Guyomard et al. 2020). Trade-offs between potentially conflicting CAP objectives must therefore be explicitly addressed.

Global food security of the EU is not a relevant argument against enhanced climatic and environmental measures in the CAP for two main reasons (Pe’er and Lakner, 2020). First, the EU is one of the largest exporters of agri-food products and one of the largest importers (notably tropical products that cannot be produced in the EU). Second, food security in the EU is far less a question of food availability than a question of food affordability, allocation, and stability that will be more efficiently addressed by public policies targeting demand aspects, in particular, access inequalities to healthier food diets (Détang-Dessendre et al. 2020). Two potential trade-offs are more relevant.

The first trade-off concerns tensions among ecological objectives. Replacing chemical inputs by nature-based practices will positively impact several local ecological dimensions (air, water, and soil quality). This can, however, occur at the expense of increased GHG emissions per kilogram of product and increased total agricultural emissions if agricultural supply (levels and composition) is maintained unchanged (whether this supply is of European or foreign origin). EU border adjustment mechanisms grounded on ecological arguments must address the international aspect of this trade-off. The national and international aspects of the trade-off require robust *in itinere* assessments of policy measures. They may also require changes in the composition of European diets, notably by reducing the consumption of animal products wherever it is excessive, for both climate and health benefits. Significantly decreasing GHG emissions of European food systems requires reducing meat consumption and livestock activity levels (Wirsenius et al. 2011; Röös et al. 2022).

The third trade-off, the most acute in the short term, is between ecological and economic impacts. Indeed, more nature-based solutions may modify production costs (fewer chemical inputs but higher labour and capital costs) and reduce crop and livestock productivities with possibly negative impacts on farm incomes that, however, will be positively impacted by higher product prices. This trade-off must not be used as an excuse to do nothing. It must be explicitly addressed. It may require corrective measures to limit the negative impacts of enhanced climatic and environmental ambitions on farm incomes. Several levers can be mobilized simultaneously: by slowing down the speed of the transition (with, however, the risk of a too slow transition); by increasing productivity through the mobilization of all productivity gain sources, including genetics and precision farming; and by developing complementary farm income sources. These complementary farm income sources can notably result from payments for ecosystem services funded by both the taxpayer and the consumer (final and intermediate) and by using savings associated with lower environmental and health damage. Compensation measures should be extended to the poorest households to ensure all access to environmentally friendly and healthy diets. However, the distribution of competences between the EU and MS poses difficulties in the coordination and implementation of these corrective measures.

### Addressing international challenges

Two international challenges are of key importance. The first concerns how WTO rules consider climatic and environmental payments that are “not limited to the extra costs or loss of income involved in complying with the government program” (WTO 1995). Such payments cannot be classified in the so-called WTO green box of agricultural subsidies authorized without limit. They fall within the amber box of non-exempted support. However, the EU remains well below its WTO amber box ceiling (Hasund and Johansson 2016), which means that the latter is not a binding constraint. Furthermore, strong arguments can be made to challenge the amber box classification in a context where the Doha Round of the WTO negotiations launched in 2001 are at a standstill.

The second related issue concerns the need to ensure a level playing field for European and foreign producers to avoid the replacement of domestic production (adjusted downwards because of increased climatic and environmental requirements) by imports from less eco-friendly countries, which would result in pollution leakages. Many studies showed that carbon leakages from de-intensifying agricultural practices in the EU could be significant (Fellmann et al. 2018; Barreiro-Hurle et al. 2021). This legitimates trade measures aimed at reducing pollution leakages. Such measures should not be limited to carbon but extended to other environmental dimensions that are global public goods, particularly biodiversity (Bellora et al. 2020). Although the EU shows laudable intentions in that domain, at least for carbon, their effective translation into bilateral and multilateral agricultural trade agreements implying the EU is lacking. It is in the EU’s interest to continue to support a rules-based international trading regime that would better take into account climatic and environmental issues.

## Conclusion

The COVID crisis and the war in Ukraine highlight the need to improve the resilience of European agricultural systems by reducing their dependency on imports supplied by a limited number of countries (direct and indirect fossil energy, animal feed ingredients). This means that any CAP reform must also be assessed from this viewpoint.

The unsustainability of European agricultural systems comes with the unsustainability of European food systems that are also unhealthy (Détang-Dessendre et al. 2020). As a result, the EGD rightfully and consistently adopts a food system approach requiring strong supply and demand public policies (EC 2020a). This means that a significantly revised CAP is essential but not sufficient. Other policies targeting agri-food trade, food losses and waste, value sharing in food chains, and diets must be implemented simultaneously (Guyomard et al. 2020).

The current European and national policies are not up to the challenges of eco-friendly, healthy, and resilient European food systems.
